# Multiple pathological fractures and muscle atrophy caused by a parathyroid carcinoma with postoperative hungry bone syndrome: A case report

**DOI:** 10.1002/cnr2.2047

**Published:** 2024-04-05

**Authors:** Qinguo Liu, Ying Zhang, Deshou Ma, Huazhen Gengzhi, Yusufu Maimaiti, Qishuai Chen, Zhijun Ma

**Affiliations:** ^1^ Clinical Medicine College Graduate School of Qinghai University Xining Qinghai China; ^2^ Department of Oncology Surgery Qinghai University Affiliated Hospital Xining Qinghai China; ^3^ Department of Head and Neck Surgery Hubei Cancer Hospital Wuhan Hubei China

**Keywords:** hungry bone syndrome, hypercalcemia, muscle atrophy, parathyroid carcinoma, pathological fracture

## Abstract

**Background:**

Parathyroid carcinoma (PC) is a rare endocrine malignancy causing pathological changes such as abnormal bone metabolism, elevated serum calcium, and impaired renal function, and uncontrollable hypercalcemia is the main cause of death in PC patients. The diagnosis of PC is challenging and relying on postoperative histopathology. Radical surgery at the first time is the only effective therapy to cure PC. Hungry bone syndrome (HBS) is a relatively uncommon complication of parathyroidectomy characterized by profound and prolonged hypocalcemia, timely electrolyte monitoring and alternative interventional protocols can prevent symptomatic hypocalcemia.

**Case:**

A 57‐year‐old man presented with multiple pathological fractures and muscle atrophy as the main symptoms accompanied by bone pain, hypercalcemia, elevated parathyroid hormone (PTH), and an enlarged left‐sided neck mass. After consultation of multidisciplinary team, he was treated conservatively with plaster bandage fixation and infusion of intravenous zoledronic acid; and then complete resection of parathyroid mass + removal of involved tissue structures + left thyroid and isthmus lobectomy + lymph node dissection in the VI region in left neck were performed. The postoperative histopathology suggested a diagnosis of parathyroid carcinoma. Calcium and fluid supplementation and oral levothyroxine tablets were given postoperatively. Unexpectedly, the patient's PTH level decreased rapidly at 24 h postoperative, and serum calcium and phosphorus decreased continuously, and he felt numb around perioral sites and fingertips, which considered to be postoperative HBS complicated by parathyroidectomy. Then, a large amount of calcium supplementation and vitamin D were given timely and the patient got better at 1 month postoperatively. At 9‐month postoperative, his bone pain and fatigue were significantly relieved compared with before with calcium, phosphorus, and PTH levels at normal range.

**Conclusion:**

The possibility of parathyroid disease, particularly PC, should be considered in the presence of multiple pathological fractures, muscle atrophy, generalized bone pain, hypercalcemia, and clear neck mass. Radical resection of the tumor lesions at the first surgery is a key element affecting the prognosis of PC, and the effective management of preoperative hypercalcemia and postoperative HBS is also of great significance for improving prognosis.

## 1 INTRODUCTION

Parathyroid carcinoma (PC) is a one of the extremely rare endocrine malignancies that accounts for approximately 0.005% of all malignancies and about 0.5% ~ 5% of cases of primary hyperparathyroidism (PHPT),^1^ causing abnormal bone metabolism, elevated serum calcium, and impaired renal function. Preoperative diagnosis of PC is challenging since there is a lack of specific clinical distinguishing features of malignant lesions, and pathology diagnosis of infiltrative growth and metastasis is considered to be the most reliable evidence for malignancy.^2^ Whole radical excision of the tumor lesion at the first surgery is the key point to cure PC.^3^ Hungry bone syndrome (HBS), a serious complication after parathyroid surgery, characterized by severe hypocalcemia following parathyroidectomy due to rapid drop of PTH after a previous long‐term elevated concentration and associated bone remineralization, usually accompanied by hypophosphatemia and hypomagnesemia.^4^, ^5^ So far, few cases of PC with muscle atrophy as the main symptom have been reported. Here, we present a PC patient with multiple pathological fractures and muscle atrophy as main symptoms and preoperative HBS complicated by parathyroidectomy.

## 2 CASE PRESENTATION

A 57‐year‐old man was admitted to the Department of Orthopedic Surgery of Qinghai University Affiliated Hospital on January 9, 2023 for generalized bone pain with fatigue for 2 years, aggravated for 1 week. He reported that systemic bone pain occurred accompanying fatigue with no obvious cause about 2 years ago, which was aggravated gradually and was not significantly relieved after taking oral analgesic drugs himself. In the past 1 week, the pain both in upper limbs and hips has been significantly worse than before. The patient had no history of trauma, weight loss, or a family history of any tumors.

Physical examinations showed that the patient was wheeled into the ward with localized pressure pain and impaired mobility in both upper limbs; equal length of both lower limbs with significant restriction of movement; positive axial percussion pain in the right lower limb; clear pressure pain with impaired movement in the right hip joint without swelling and bruising under the skin; the muscles of the extremities were atrophied to varying degrees, the muscle strength of forearms of two upper limbs, and two lower limbs was about grade 3; the biceps reflex, triceps reflex, and knee tendon reflex were weakened; no sensory abnormalities in the extremities; the pathological reflex was not elicited; meningeal irritation was negative; there was a 2 cm × 2 cm palpable mass, which is hard and has poor mobility in the left neck; and no significantly enlarged lymph nodes palpated.

Laboratory tests showed his serum calcium level was at 3.55 mmol/L (normal range: 2.25–2.75 mmol/L), alkaline phosphatase (ALP) was at 608 U/L (normal range: 40 ~ 125 U/L), creatinine (Cr) was at 110 mmol/L (normal range: 57–97 mmol/L), urea nitrogen (UN) was at 8.7 mmol/L (normal range:2.5 ~ 7.1 mmol/L), parathyroid hormone (PTH) was at 2565.0 pg/mL (normal range: 15–65 pg/mL), and there were no obvious abnormalities in his thyroid function tests and other tests.

**FIGURE 1:**
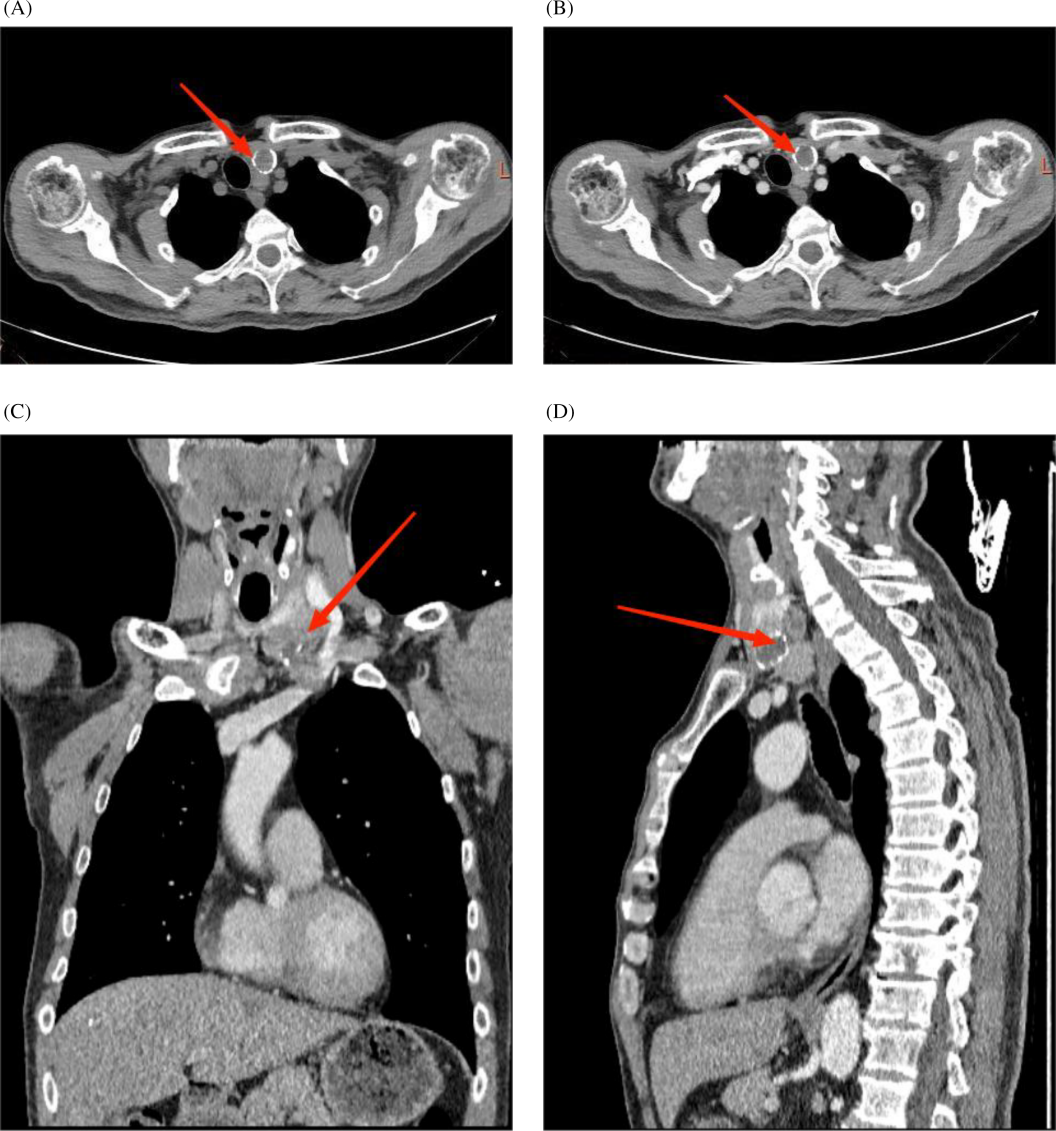
Neck computed tomography (CT) and contrast enhanced computed tomography (CECT) scan: (A) CT shows an enlarged left thyroid lobe within an irregular slightly hypointense shadow and circular calcification foci and a slightly rounded hypointense shadow posterior to the left inferior thyroid lobe; (B) CECT shows enhanced scanning of low‐density shadows of the left thyroid lobe; (C) coronal view of the thyroid shows an irregularly shaped left cervical mass protruding downward into the left paratracheal space and compressing the jugular vein; (D) sagittal view of the left thyroid lobe shows the papillary mass growing backward into the anterior esophageal space.

Neck ultrasound (US) showed a 3.5 cm × 3.2 cm hypoechoic mass with tiny calcified dots in the left thyroid gland and a 1.5 cm × 1.5 cm hypoechoic nodule posterior to the left inferior thyroid lobe with undefined margins, considering a possibility of parathyroid origin; neck computed tomography (CT) and contrast‐enhanced CT scan showed an enlarged left thyroid lobe within a 3.4 cm × 2.8 cm irregular slightly low‐density shadow and circular calcification foci protruding downwards into the left paratracheal space; and a 1.3 cm slightly rounded low‐density shadow posterior to the left inferior thyroid lobe grows backward into the anterior esophageal space, considering a parathyroid mass (Figure 1).

**FIGURE 2:**
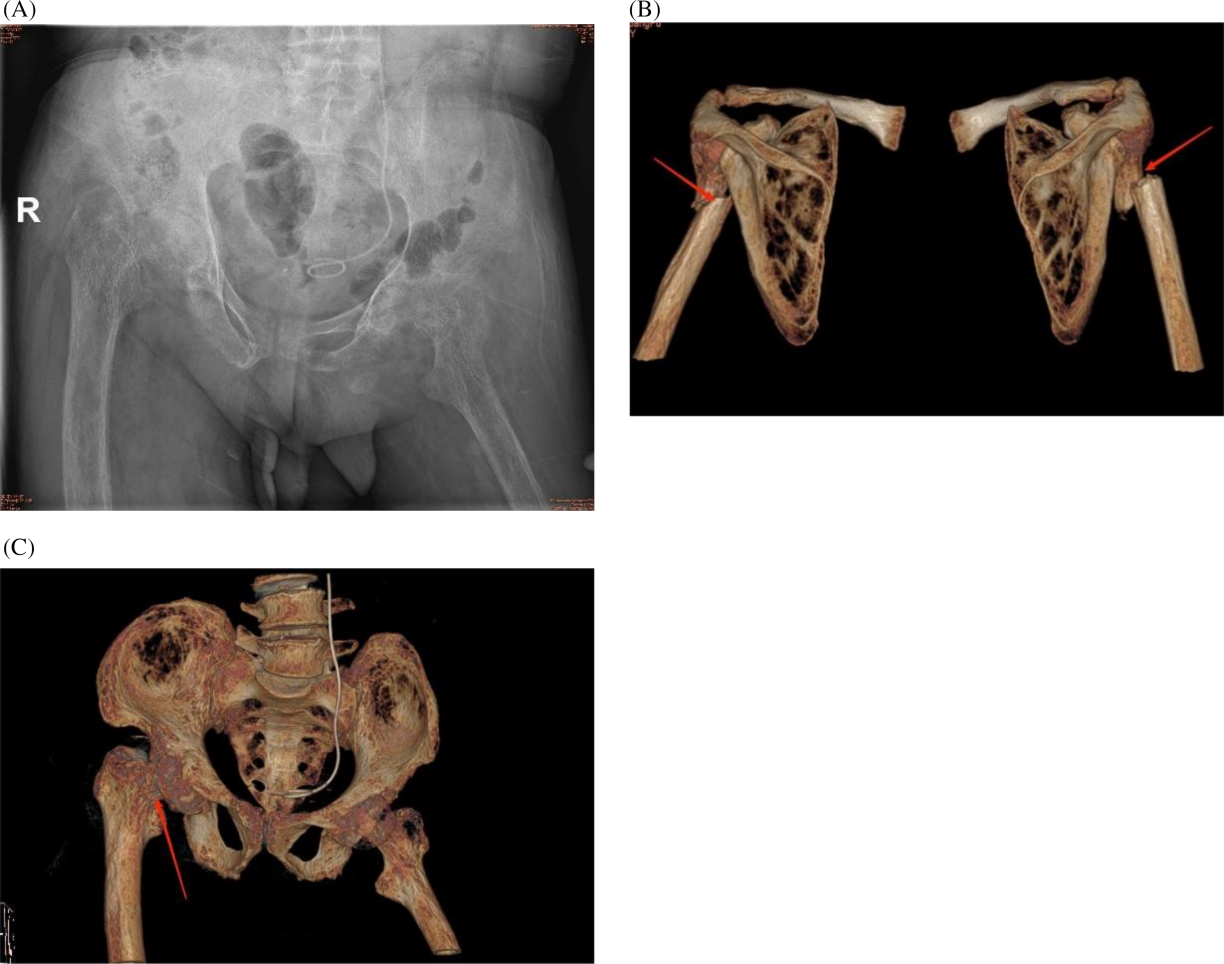
(A) X‐ray of the pelvis, showing a right femoral neck fracture and muscle atrophy around the left hip; (B) three‐dimensional computed tomography (CT) of the shoulder joints, presenting bilateral humeral shaft fractures; (C) three‐dimensional CT of the pelvis, presenting the fracture of the right femoral neck which alignment is good.

X‐ray of pelvis showed a right femoral neck fracture and muscle atrophy around the left hip (Figure 2A). Three‐dimensional CT scan of the shoulder joint and pelvis showed the bilateral humeral shaft fractures, the right femoral neck fracture, and the decreased bone mineral density both of the shoulder joints and pelvic constituent bones (Figure 2B,C). Abdomen CT scan showed multiple intrapelvic stones in both kidneys.

Given the patient had significantly high calcium levels (3.55 mmol/L) and PTH (2565.0 pg/mL), elevated UN (8.7 mmol/L) and Cr (110 mmol/L), a neck mass and severe skeletal damage, the departments of endocrinology, orthopedics, nephrology, neurology, imaging, and oncology surgery were invited for multidisciplinary team (MDT). MDT suggested that in view of severe osteoporosis and thin bone cortex, the patient could not undergo a fracture internal fixation surgery; the bilateral humeral shaft fractures and the right femoral neck fracture without significant displacement could be treated conservatively such as plaster bandages, analgesic, infusion of intravenous zoledronic acid (4 mg) and taking calcitriol capsules orally; in addition, the possibility of parathyroid tumor was not excluded, so the surgery for thyroid was recommended.

**FIGURE 3:**
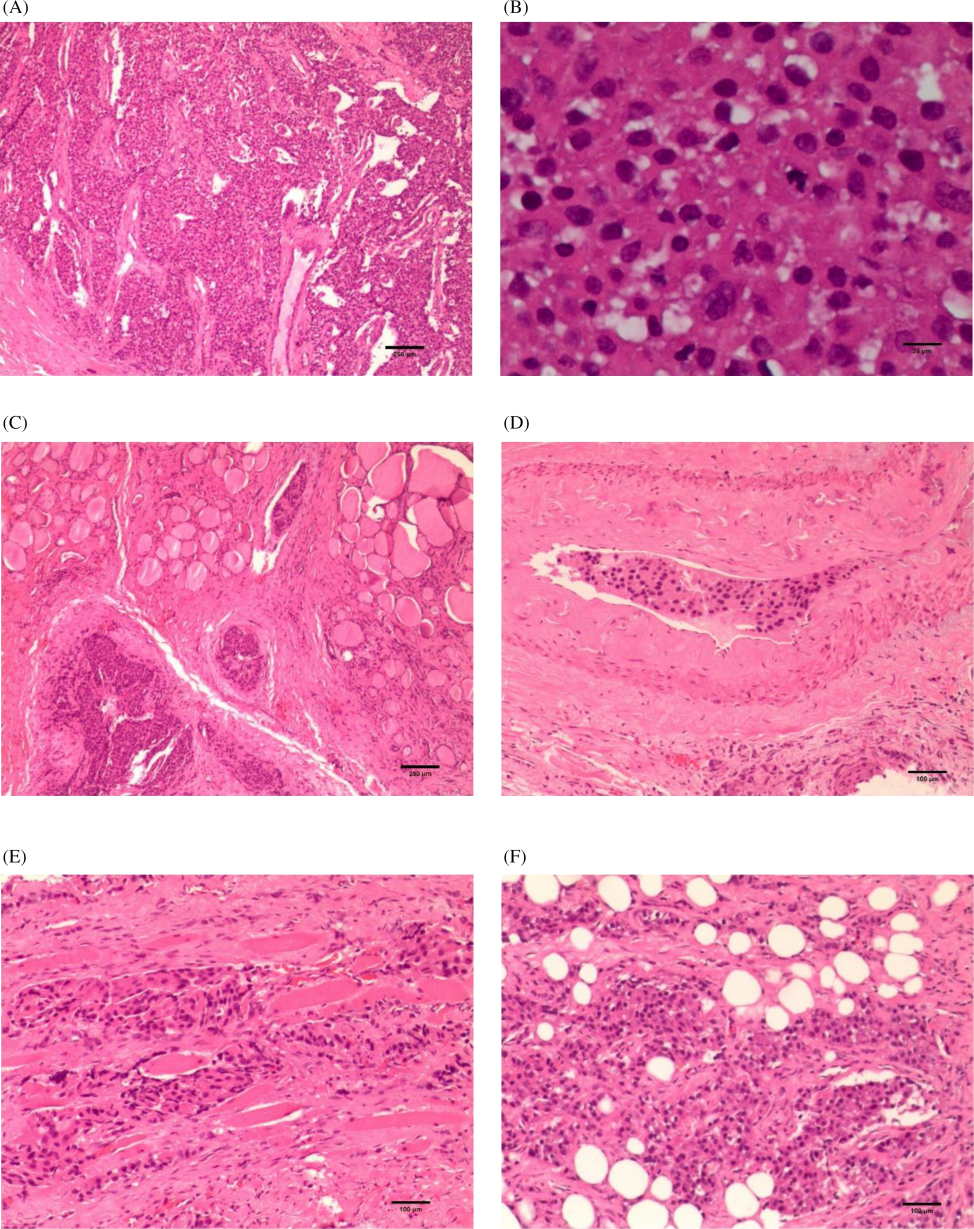
Hematoxylin & eosin staining: (A) is parathyroid carcinoma tumor cell (scale bar = 250 μm, ×40); (B) is nuclear division of tumor cell (scale bar = 25 μm, ×400); (C) is thyroid tissue invasion of parathyroid carcinoma (scale bar = 250 μm, ×40); (D) is vascular invasion (scale bar = 100 μm, ×100); (E) is striated muscle tissue invasion of parathyroid carcinoma (scale bar = 100 μm, ×100); (F) is adipose tissue invasion of parathyroid carcinoma (scale bar = 100 μm, ×100).

**FIGURE 4:**
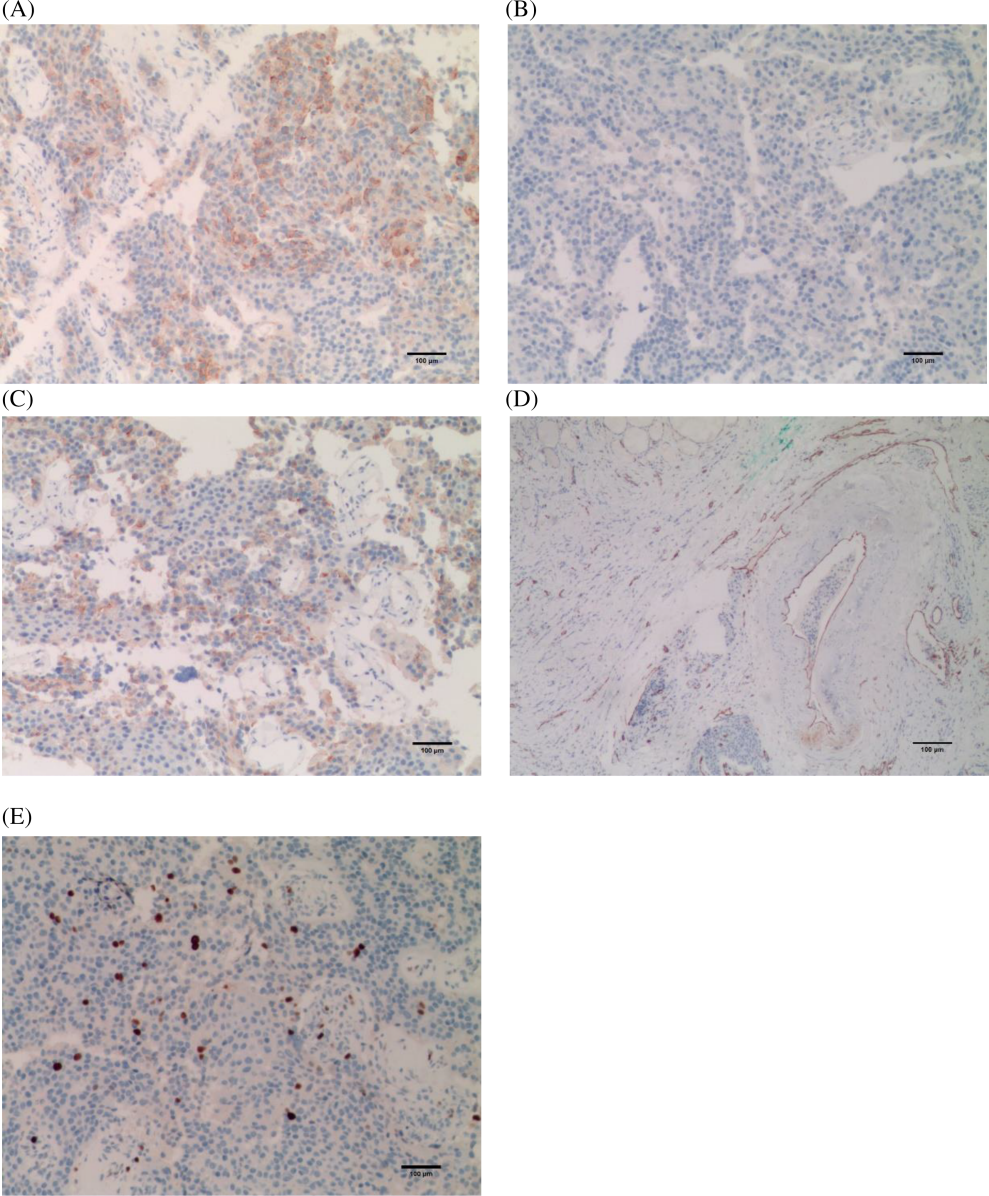
Immunohistochemical staining: (A) tumor cells are positive for PTH (scale bar = 100 μm, ×100); (B) tumor cells are negative for thyroid transcription factor‐1 (TTF‐1) (scale bar = 100 μm, ×100); (C) tumor cells are partially positive for synaptophysin (SYN) (scale bar = 100 μm, ×100); (D) tumor cells are positive for CD31 (vascular expression) (scale bar = 100 μm, ×100); (E) Ki67 is at 10%(scale bar = 100 μm, ×100).

Complete resection of the left parathyroid mass was performed on January 18, 2023 after completing related examinations such as bone scan to exclude primary and secondary bone malignancies. Intraoperatively, left parathyroid mass invading the left thyroid lobe and surrounding tissues was seen, and freezing results were considered as a tumor of parathyroid origin, then radical resection of parathyroid tumor + removal of involved tissue structures + left thyroid and isthmus lobectomy + lymph node dissection in the VI region of the left neck were performed. Postoperative pathology was diagnosed as PC with no lymph node metastasis (0/4). Hematoxylin & eosin (HE) staining showed that PC invaded the thyroid lobe, fat, and striated muscle tissues (Figure 3). Immunohistochemically, tumor cells were negative for thyroid transcription factor‐1 (TTF‐1), negative for thyroglobulin, negative for Chromogranin A (CgA), partially positive for synaptophysin, positive for PTH, positive for CD31 and CD34 (vascular expression), positive for D2‐40 (lymphatic+), Ki67 was at 10% (Figure 4). Calcium and fluid supplementation and oral levothyroxine tablets were given postoperatively.

At 24 h postoperatively, the patient's blood pressure was at 100/70 mmHg; heart rate was 120 beats per minute; the PTH dropped to 9.57 pg/mL rapidly; calcium level reduced to 2.88 mmol/L and phosphorus level lowered to 0.55 mmol/L; then a large amount of calcium gluconate and vitamin D were given timely. Unexpectedly, his serum calcium and phosphorus levels continued to decline in 1 week postoperatively while PTH gradually increased and returned to the normal range (Table 1); in the meantime, he was in poor mental condition, and felt tired and numb in perioral sites and fingertips; and we considered this condition to be HBS. Calcium supplementation was given continually for 1 month, and the patient got better with numbness of perioral sites and fingertips disappearing; and the rechecked serum calcium level was at 2.15 mmol/L and PTH was at 76.80 pg/mL (Table 1) on February 21, 2023. Nine months following parathyroidectomy (October 25, 2023), the patient's systemic bone pain and fatigue were distinctly relieved compared with before with the calcium and PTH decreased to the normal range on rechecking (Table 1). His fractures have reached the clinical healing standard, and the external fixation has been released. He can move freely and do some low intensity physical work.

**TABLE 1:** Pre and postoperative changes of PTH, calcium, and phosphorus levels in the patient.

Time	PTH (15–65 pg/mL)	Ca (2.11–2.51 mmol/L)	P (0.85–1.51 mmol/L)
Preoperation	2565.00	3.55	0.83
24 h	9.57	2.88	0.55
48 h	19.50	2.22	0.36
72 h	36.20	2.03	0.52
96 h	42.32	1.90	0.31
5‐day	57.70	1.82	0.41
7‐day	69.12	1.60	0.40
8‐day	89.66	1.74	0.73
1‐month	76.80	2.15	0.84
3‐month	73.45	2.35	0.89
6‐month	68.00	2.33	0.96
9‐month	36.50	2.35	0.95

Abbreviation: PTH, parathyroid hormone.

## 3 DISCUSSION

PC is an extremely rare endocrine malignancy and its etiology is incompletely understood. Most PC patients are often present sporadically while some cases are associated with hereditary disorders that cause PHPT, such as hyperparathyroidism‐jaw tumor syndrome (HPT‐JT), multiple endocrine neoplasia types 1 and 2, and familial isolated hyperparathyroidism.^6^ HPT‐JT is an autosomal dominant disorder, and PC may occur in approximately 20% of HPT‐JT cases.^7^ Mutations of the cell division cycle 73 (CDC73) gene has been reported as the most common tumor suppressor gene associated with PC and the absence of parafibromin caused by mutations of CDC73 makes parathyroid tissue more susceptible to carcinogenesis.^3^, ^6^ In addition, studies have also suggested that repeated mutations of the Prune Homolog 2 with BCH Domain (PRUNE2) gene and AarF Domain‐Containing Kinase 1 (ADCK1) gene, genetic amplification of Cyclin D1 gene, and abnormal activation of PI3K/AKT/mTOR signaling pathway are related to the occurrence of PC.^6^, ^8^ In this case, there were not any abnormalities found in the above common genes' detection, and the specific etiology of this patient still needs to further study.

The clinical manifestations of PC are associated with hypercalcemia and markedly elevated PTH, which are nonspecific, making it difficult to distinguish from benign lesions (adenomas and hyperplasia) that cause PHPT. Nevertheless, there have been reports that some clinical features may be useful to predict malignancy: symptomatic hypercalcemia (calcium levels above 3.35 mmol/L with renal impairment, urinary system stones, bone pain, pathological fractures and other signs and symptoms of renal and skeletal system involved), high levels of PTH (>800 pg/mL or more than 5 ~ 10 times the upper normal value), parathyroid crisis and palpable neck mass or signs of adjacent soft tissue invasion at ultrasonography.^1^, ^8^, ^9^ In the presented case, the patient had severe osteoporosis with multiple pathological fractures as the main symptom, accompanied by generalized bone pain and fatigue and a palpable neck mass (2 × 2 cm); laboratory tests showed elevated serum calcium, elevated PTH to nearly 40 times the upper normal value, elevated ALP, creatinine, and urea nitrogen levels as well as abdominal CT showed urological stones, and given the strong clinical evidence described above, we infer a high likelihood of malignancy. Due to the lack of routine screening of serum calcium and PTH in China, asymptomatic hypercalcemia caused by PHPT is difficult to detect and most parathyroid tumors are diagnosed only after the occurrence of generalized bone pain and pathological fractures caused by hypercalcemia. Additionally, pathological fractures and multiple bone damage are often misdiagnosed as malignancies such as metastatic bone tumor and giant cell tumor of bone due to osteolytic lesions at bone pain and fracture sites. These easily misdiagnosed malignancies can be excluded by above laboratory tests and imaging features. After performing relevant examination such as bone scan to rule out primary and secondary bone malignancies, the diagnosis of parathyroid tumor was considered comprehensively with high possibility of malignancy; and the diagnosis of PC confirmed by postoperative pathology.

The study has shown that the common clinical symptoms of PC are listed according to their prevalence: skeletal manifestations > renal manifestations > fatigue > palpable neck mass > neuropsychiatric symptoms.^3^ As a result, muscle‐related symptoms are often overlooked or misdiagnosed. Nevertheless, muscle atrophy may be a major clinical manifestation of PC. PC has an insidious onset, long‐term hypercalcemia also leads to decreased muscle strength, especially the proximal parts of the limbs; and some patients present muscle pain and muscle atrophy.^3^, ^10^ In addition, PTH also regulates the metabolism of fat and muscle tissues. Studies have shown that PTH and parathyroid hormone‐related peptides (PTHrPs) interact with PTH receptors to activate protein kinase A (PKA) and induce the expression of thermogenesis‐related gene uncoupling protein‐1 (UCP1) and atrophy‐related genes such as atrogin‐1 and MuRF1, causing adipose tissue browning and loss and muscle atrophy; PTH and PTHrP can also activate the ubiquitin–proteasome system, resulting in muscle protein degradation, muscle mass and muscle strength loss.^10^, ^11^, ^12^ In this case, the patient's muscle of limbs was atrophied to varying degrees, combing physical examinations with other symptoms and imaging findings can exclude the possibility caused by neurological lesions and consider the possibility caused by PC.

Imaging tests such as US and CT are commonly used for preoperative localization of parathyroid neoplasms, whereas the definitive diagnosis of PC only relies on pathological analysis. WHO regards histologic evidence of infiltrative growth including capsule and extra‐capsule extension and vascular invasion as minimum criteria of diagnostic malignancy; high mitotic activity (usually above five mitoses per 50 high‐power fields), pathological mitotic figures, prominent nucleoli, broad fibrous septa, and tumor necrosis are considered morphological evidence for malignancy.^2^ In addition, immunohistochemistry is necessary to confirm the diagnosis of PC. PTH, neuroendocrine tumor markers SYN, and CgA are helpful in the diagnosis of PC; when the Ki‐67 index is greater than 5%, it is alert to consider the possibility of malignancy.^3^ In this case, the tumor tissue was heavily fibrous‐segregated and the tumor cells were heterogeneous with obvious nuclei and pathological mitotic figures; the tumor invaded the thyroid and surrounding soft tissues, and necrosis and intravascular tumor embolus were seen; immunohistochemically, tumor cells were negative for TTF‐1, thyroglobulin, CgA, partially positive for synaptophysin, positive for PTH, CD31, and CD34 (vascular expression), positive for D2‐40 (lymphatic+), and Ki67 was 10%; the above results are consistent with diagnosis of PC.

Radical resection of the tumor lesions at the first surgery is the only chance to cure PC potentially.^3^ Up to now, there is no evidence to show survival benefits from chemotherapy, radiotherapy and immunotherapy in the treatment of PC.^3^, ^13^ Most PC patients will significantly improve their life quality after operation with bone pain, muscle weakness, fatigue and other symptoms being relieved, and recover from severe osteoporosis effectively. HBS is a rare postoperative complication in some patients with PHPT, and the mechanism is the action of osteoclasts after radical resection of PC.^5^, ^14^ After radical resection of PC, the sudden decrease of PTH, which acts on bone resorption of osteoclasts does not affect the process of osteogenesis of osteoblasts; osteoclasts activity is replaced by osteoblasts activity, resulting in a rapid increase of calcium uptake, which leads to a large amount of serum calcium into the bone and deposit in the bone tissues, inducing severe symptomatic hypocalcemia such as numbness of perioral sites and fingertips, tetany, and even dyspnea. Studies have been reported currently that risk factors of occurrence of HBS include high PTH with a previous long‐term elevated concentration, high calcium levels, and excessive ATP elevation, and so forth.^5^, ^15^, ^16^ Active preoperative measures may reduce the incidence of postoperative HBS, Davenport and Stearns reported that preoperative pamidronate in combination with high doses of oral alfacalcidol and calcium supplements can prevent postoperative symptomatic hypocalcemia and help to prevent the occurrence of HBS.^17^ In our case, the patient was given intravenous zoledronic acid combined with oral calcitriol before the operation in order to prevent the occurrence of HBS after parathyroid surgery since he had the above high‐risk factors of postoperative HBS. However, it was still not effective in avoiding the occurrence of HBS, then he gradually recovered after large amounts of supplementation of calcium gluconate and vitamin D in 1 month.

The prognosis of PC is poor, and hypercalcemia and metabolic complications of hypercalcemia are the leading causes of its death, for the 5‐year survival after surgery is 60% ~ 93%.^3^ Even after radical resection of the tumor, the 5‐year recurrence rate of PC is still as high as 33% ~ 82%.^1^, ^3^ For the recurrent or metastatic lesions, the tumor burden can be reduced by various means such as surgery and intervention therapy; calcium‐lowering measures such as loop diuretics, calcitonin, and intravenous bisphosphonates should be actively used to manage hypercalcemia, when the tumor lesions are no longer suitable for surgical resection; and monitoring calcium closely is necessary to extend survival. In this case, after more than 9 months of postoperative follow‐up, the patient's general condition got better with no sign of recurrence or metastasis, but further follow‐up is still needed to assess the progression of the disease.

In conclusion, we report a male patient with generalized bone pain, multiple pathological fractures, hypercalcemia, neck mass, and muscle atrophy, in whom HBS was diagnosed 1 week after operation and the diagnosis of PC was confirmed by postoperative histopathology. The diagnosis of PC in the case was complex and challenging as its clinical presentations were not characteristic, making it difficult to differentiate preoperatively from parathyroid adenoma or hyperplasia, which also cause PHPT. However, the strength of the clinical evidence helped us to suspect malignancy early. When one presents with generalized bone pain, multiple pathological fractures, hypercalcemia, muscle atrophy and a palpable mass in the neck, we should consider the possibility of parathyroid disease, especially PC. Muscle atrophy is relatively rare compared with other manifestations of PC, and we should take it seriously. In addition, HBS is a rare and life‐threatening complication of parathyroidectomy; timely electrolyte monitoring and alternative interventional protocols can prevent symptomatic hypocalcemia and improve the prognosis. Nevertheless, how to determine the appropriate timing of introducing calcium supplementation therapy in PC patients with high‐risk factors for developing HBS deserves further investigation.

## AUTHOR CONTRIBUTIONS

Deshou Ma was the surgeon. Qinguo Liu and Ying Zhang analyzed and interpreted the patient data regarding parathyroid carcinoma and secondary hyperparathyroidism. Qinguo Liu drafts the manuscript. Huazhen GengZhi, Deshou Ma, Qishuai Chen, and Zhijun Ma were involved in the preparation of the manuscript. Zhijun Ma and Yusufu Maimaiti reviewed and verified the content of the manuscript. All authors have read and approved the final manuscript.

## FUNDING INFORMATION

This case report was funded by the National Natural Science Foundation of China (82060485) and the National Natural Science Foundation of China (82160570).

## CONFLICT OF INTEREST STATEMENT

The authors have stated explicitly that there are no conflicts of interest in connection with this article.

## ETHICS STATEMENT

All methods were carried out in accordance with relevant guidelines and regulations. The patient provided written informed consent for data collection.

## CONSENT FOR PUBLICATION

Written Informed consent was also obtained from the patient for publication of this case report. “I understand that the text and any pictures published in the article will be freely available on the internet and may be seen by the general public.”

## Data Availability

The datasets used and analyzed during the current study are available from the corresponding author on reasonable request via mzjfamai@163.com.
